# Impact of Autoantibodies against Glycolytic Enzymes on Pathogenicity of Autoimmune Retinopathy and Other Autoimmune Disorders

**DOI:** 10.3389/fimmu.2017.00505

**Published:** 2017-04-28

**Authors:** Grazyna Adamus

**Affiliations:** ^1^School of Medicine, Casey Eye Institute, Oregon Health and Science University, Portland, OR, USA

**Keywords:** autoantibodies, autoimmune diseases, retinopathy, glycolysis, enzymes characterization

## Abstract

Autoantibodies (AAbs) against glycolytic enzymes: aldolase, α-enolase, glyceraldehyde-3-phosphate dehydrogenase, and pyruvate kinase are prevalent in sera of patients with blinding retinal diseases, such as paraneoplastic [cancer-associated retinopathy (CAR)] and non-paraneoplastic autoimmune retinopathies, as well as in many other autoimmune diseases. CAR is a degenerative disease of the retina characterized by sudden vision loss in patients with cancer and serum anti-retinal AAbs. In this review, we discuss the widespread serum presence of anti-glycolytic enzyme AAbs and their significance in autoimmune diseases. There are multiple mechanisms responsible for antibody generation, including the innate anti-microbial response, anti-tumor response, or autoimmune response against released self-antigens from damaged, inflamed tissue. AAbs against enolase, GADPH, and aldolase exist in a single patient in elevated titers, suggesting their participation in pathogenicity. The lack of restriction of AAbs to one disease may be related to an increased expression of glycolytic enzymes in various metabolically active tissues that triggers an autoimmune response and generation of AAbs with the same specificity in several chronic and autoimmune conditions. In CAR, the importance of serum anti-glycolytic enzyme AAbs had been previously dismissed, but the retina may be without pathological consequence until a failure of the blood–retinal barrier function, which would then allow pathogenic AAbs access to their retinal targets, ultimately leading to damaging effects.

## Introduction

**Figure 1:**
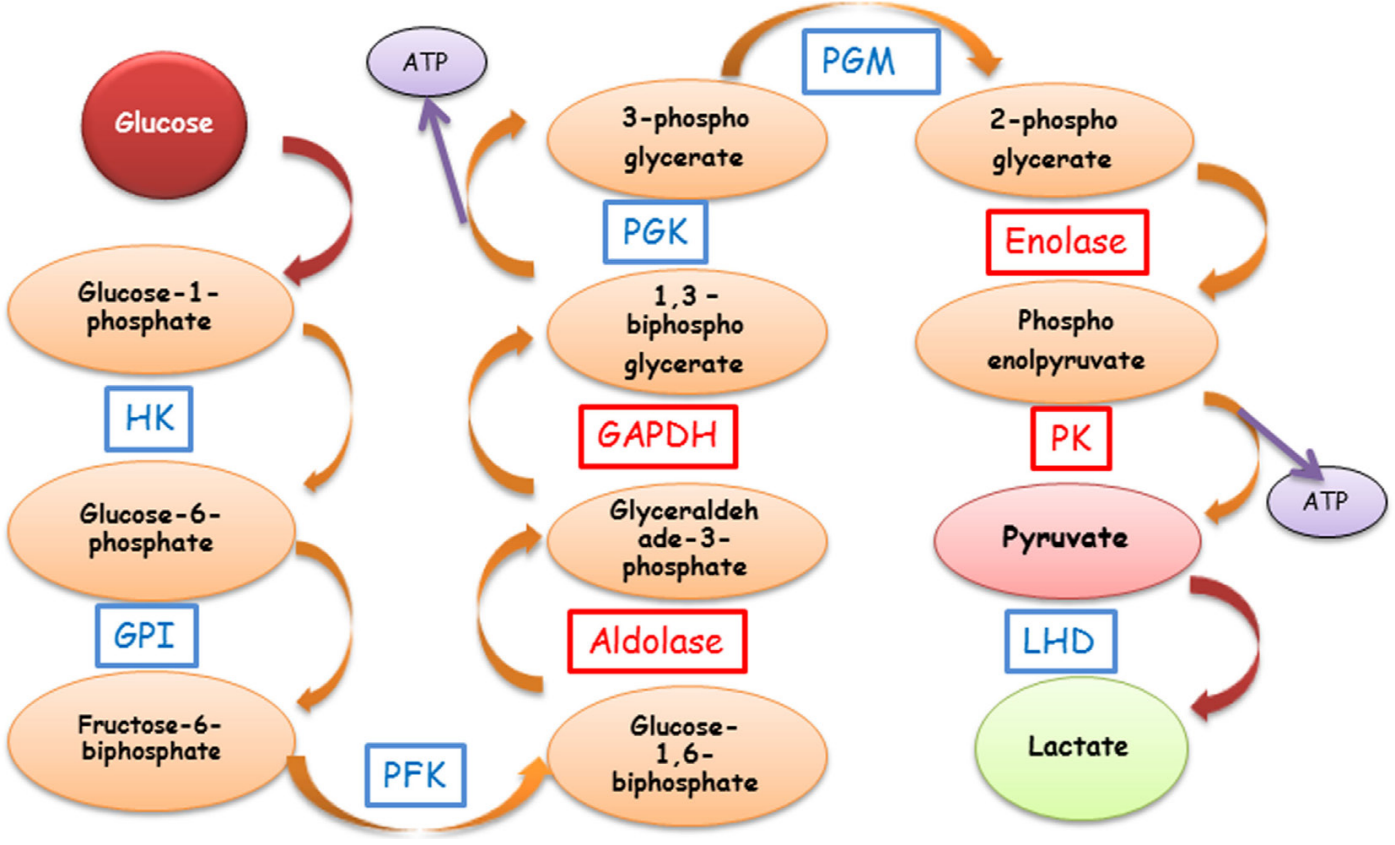
**Schematic representation of the glycolytic pathway and anti-enzymatic protein autoantibody involvement**. The most common AAbs are against GAPDH, aldolase, enolase, and PK (red boxes). Abbreviations: GAPDH, glyceraldehyde-3-phosphate dehydrogenase; GPI, glucose-6-phosphate isomerase; HK, hexokinase; LDH, lactate dehydrogenase; PFK, phosphofructokinase; PGK, phosphoglycerate kinase; PGM, phosphoglycerate mutase; PK, pyruvate kinase; ATP, adenosine triphosphate; AAbs, autoantibodies.

Humans are genetically diverse, but despite their immunological differences, they often produce autoantibodies (AAbs) against similar autoantigens. In particular, a number of studies have shown that α-enolase and other glycolytic enzymes are targeted by AAbs associated with various pathological conditions, including autoimmune retinopathy (AR) (1–6). It is surprising because these enzymes play an important role in the cellular production of energy through the glycolytic pathway, which metabolizes glucose to pyruvate in a chain of enzymatic reactions to produce cellular adenosine triphosphate (ATP) (Figure 1). Energy needs of a given cell type depend on tissue physiology, in particular, metabolically active retinal photoreceptor cells that have great energetic requirements (7–10). Thus, blocking cellular functions of glycolytic enzymes by pathogenic AAbs could be devastating to any cells’ survival.

**Figure 2:**
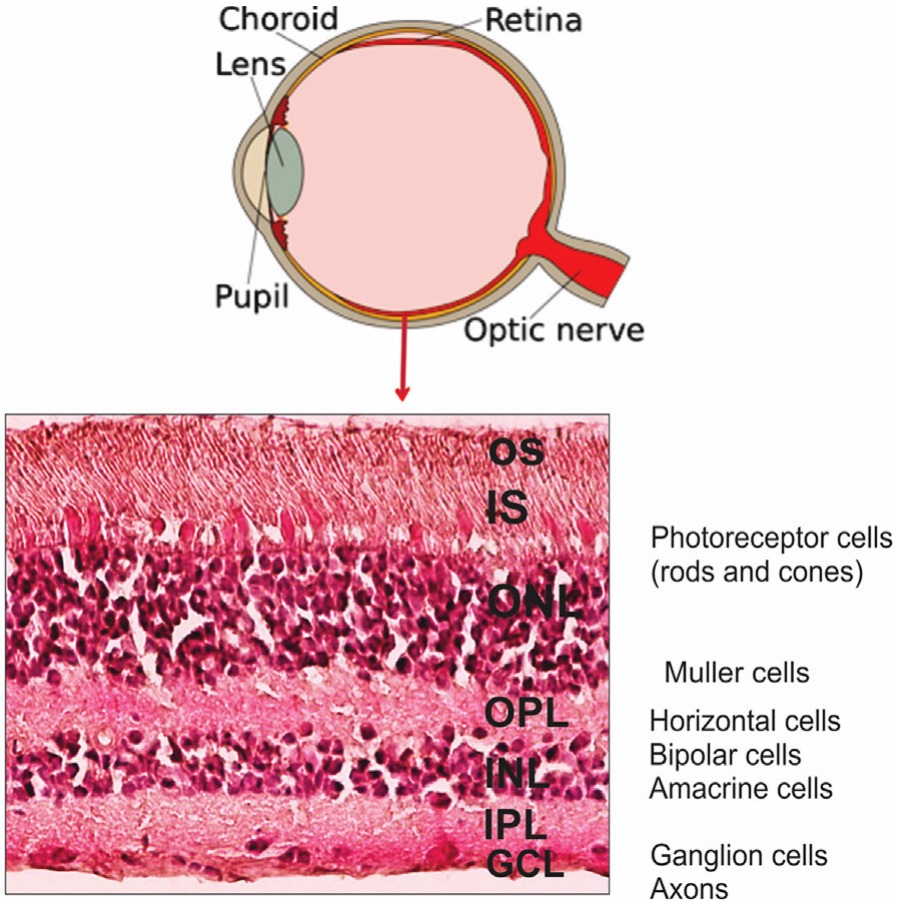
**Anatomy of human eye and retinal layers**. Diagram of the eye and the organization of retinal layers and cells showing H&E staining of representative section of human retina. Abbreviations: OS, outer segments; IS, inner segments; ONL, outer nuclear layer; OPL, outer plexiform layer; INL, inner nuclear layer; IPL, inner plexiform layer; GCL, ganglion cell layer.

The retina is a light-sensitive tissue located in the back of the eye, which is composed of layers of neurons such as photoreceptors, bipolar cells, and ganglion cells, supported by glial cells (Müller cells and astrocytes) (11). It is protected behind blood-ocular barriers (11). The cross-section of retinal layers is shown in Figure 2. The retina converts photons of light into electric signals and sends them to the brain in the process called the visual transduction cascade. This process takes place in the outer segments (OS) of the photoreceptors, including the cell membranes and pigment disk membranes of the OS. There are two types of photoreceptor cells: the rods and cones. Rods are responsible for black-and-white vision, while cones support the color vision (12). Glycolysis occurs in photoreceptor and Müller cells, providing energy to photoreceptors cells (13–15). Glucose reaches photoreceptor cells (the outer retina) from the blood, through the pigment epithelium cell layer (RPE) by glucose transmembrane transporter GLUT1 (16). Thus, RPE is important in this process by providing metabolic support to photoreceptor cells (17). In addition to energy production, the glucose metabolism may be involved in photoreceptor cell death and survival, suggesting that glycolysis and apoptosis are linked (18, 19).

Glycolytic enzymes are evolutionarily conserved proteins, have multiple functions in the cell not related to glycolysis, and are highly autoimmunogenic (19, 20). The enzymes are not only present intracellularly, but also on the cell surface, including the surface of neuronal cells; therefore, they are conceivably being exposed to the immune system (21). AAbs against four glycolytic enzymes: aldolase (ALDO), α-enolase (ENO1), glyceraldehyde-3-phosphate dehydrogenase (GAPDH), and pyruvate kinase M2 (PKM2) are particularly prevalent in sera of patients with autoimmune retinopathies, including cancer-associated retinopathy (CAR) and melanoma-associated retinopathy (MAR). Similar AAbs were also reported in autoimmune uveitis (22, 23). Although anti-enolase and other enzymes can be detected in low titers in some healthy individuals (from 0 to 10% depending on population studied), they were likely generated in response to common infections (18, 24). In contrast, AAb titers in disease are always found to be significantly higher (25).

Cancer-associated retinopathy is a rare retinal disorder, and its prevalence is unknown, but more patients with CAR or CAR-like symptoms (AR) have been identified each year (26). In CAR, patients developed a sudden loss of vision, visual field defects, photopsias, color vision loss, and dysfunction of rod and/or cone responses in the presence of a remote cancer and serum anti-retinal AAbs. These were, possibly, generated in response to antigens released from tumor, including glycolytic enzymes captured by antigen-presenting cells (27–29). To understand the role of glycolytic enzymes as target autoantigens, it is important to appreciate their biological significance in retinal disease and other autoimmune conditions. This review is an attempt to explain the high frequency of AAbs against glycolytic enzyme association in autoimmune diseases.

## Enolase

Enolase is the most frequent autoantigen in autoimmune retinopathies, including CAR and MAR (24, 30). Of all patients with anti-retinal antibodies, over 30% have anti-α-enolase antibodies (31). Furthermore, anti-enolase AAbs have been reported in cancer, several systemic autoimmune disorders, connective tissue disorders, and inflammatory diseases, including Behçet’s disease, Hashimoto’s encephalopathy, ANCA-positive vasculitis, rheumatoid arthritis (RA), systemic lupus erythematosus (SLE), multiple sclerosis (MS), primary sclerosing cholangitis, and inflammatory bowel disease (3–5, 32–40).

Enolase exists as homo- or hetero-dimers of three subunits, α, β, and γ, all of which are targets of autoimmunity. α-Enolase encoded by the gene ENO1 is ubiquitous, β-enolase encoded by the gene ENO3 is as a structural protein in the lens of some species, and possibly a suppressive cytokine, and γ-enolase encoded by the gene ENO2 is a neuron-specific enolase (37, 41). This enzyme catalyzes a formation of phosphoenolpyruvate from 2-phosphoglycerate in glycolysis (Figure 1). Enolase-α is present throughout the retina, including OS of photoreceptor cells and Müller cells (24, 42, 43).

Enolase acts as a plasminogen receptor, modulating pericellular fibrinolytic activity, as well as other non-glycolytic functions, resulting from its subcellular and membrane localizations (44). Such a differential expression of α-enolase in tissues/organs has been linked to several pathologies, such as cancer, Alzheimer’s disease (AD), and RA, among others (29, 37, 45). In addition, α-enolase has been detected on the surface of endothelial cells, hematopoietic cells (monocytes, T cells, and B cells), and neuronal cells (44). The surface expression of α-enolase in various eukaryotic cells has been found to be dependent on the pathophysiological conditions of a given cell (46). The upregulation of enolase, during metabolic processes, and its release from dying cells may also lead to its uptake by antigen-presenting cells. The subsequent B cell activation could trigger an excessive production of anti-enolase AAbs that can potentially initiate tissue injury, e.g., as a result of immune complex deposition (Figure 2).

In recent retinal research, remarkable findings were reported on the interaction between proteins involved in phototransduction and glycolysis. Smith et al. demonstrated a direct binding between retinal arrestin and retinal enolase, showing that arrestin slowed the catalytic activity of enolase and the light-driven translocation of arrestin modulated metabolic activity of photoreceptors (42). In contrast to arrestin, α-enolase does not change its location in the photoreceptor cell in response to light. In the dark-adapted retina, enolase was found to co-localize with arrestin in the inner segments (IS) and outer nuclear layer (ONL), but remained in the IS when arrestin translocated in response to light adaptation. These findings may explain an occasional detection of anti-enolase and anti-arrestin AAbs in the same patient with CAR or MS, suggesting that such complexes could be released from damaged photoreceptor cells, then processed by antigen-presenting cells (38, 47). Surprisingly, AAbs against arrestin and α-enolase were also reported in some patients with coronary heart disease (48). When investigators exposed cardiomyocytes to monoclonal antibody against arrestin or enolase *in vitro*, they observed decreased cell proliferation, suggesting that antibodies bound to the membrane-exposed epitopes of arrestin and enolase in living cells (48). The study suggested that these AAbs may be involved in the induction of cardiac autoimmune diseases.

Certain properties of α-enolase, especially those related to surface exposure and plasminogen-binding, suggest its role in the initiation of a disease process by modulating the pericellular and intravascular fibrinolytic system (49). Enolase is translocated from the cytoplasm to the cell surface and then serves as the plasminogen receptor on the surface of various cells and enhances pericellular plasmin production for cell invasion (50). The enolase epitope involved in the plasminogen-binding of α-enolase is located within the amino acid sequence 257–272. This binding site is different from the main pathogenic epitope in patients with CAR (the sequence 56–63), and patients with endometriosis (the sequences 53–87 and 207–238), or healthy individuals, suggesting that these disorders are not associated with disturbances of the intravascular and pericellular fibrinolytic system (1, 3, 51, 52). The linking of epitopes to specific autoimmune and inflammatory conditions is a challenge, so more research is needed to decide whether enzymatic protein epitopes would better distinguish their association with a particular disease and have a causative activity.

## Glyceraldehyde-3-Phosphate Dehydrogenase

The next common autoantigen in disease is GAPDH, which catalyzes the oxidative phosphorylation of glyceraldehyde-3-phosphate to 1,3-biphosphoglycerate, leading to the reduction of nicotinamide adenine dinucleotide (NAD^+^) to NADH during glycolysis (Figure 1). GAPDH also has various functions outside the glycolysis that are related to its localization in cytoplasm and nucleus. These functions include the cell cycle, nuclear tRNA export, DNA replication and repair, endocytosis, exocytosis, cytoskeletal organization, iron metabolism, carcinogenesis, and cell death (53–57). It has been suggested that this functional diversity may be regulated by posttranslational GAPDH modification, by subcellular protein–protein interaction, or by protein–nucleic acid interactions (54). The first indication related to its non-glycolytic function was GAPDH association with non-glycolytic protein tubulin and its ability to bundle microtubules (58).

Autoantibodies against GADPH have been found in sera of patients with autoimmune retinopathies and optic neuropathies, including patients with paraneoplastic syndrome, and in MS as an autoimmune response to neurons and axons (59–61). In photoreceptor cells of the retina, GAPDH is distributed throughout the cell, as well as in the plasma membrane of the rod outer segments (ROS), and consists of about 2% of total ROS proteins and more than 11% of total plasma membrane proteins (62). Like α-enolase, GADPH does not translocate upon exposure of the retina to light, but it is involved in translocation of the rod phototransduction protein α-transducin from the rod inner to OS during dark adaptation (63). Both proteins GAPDH and α-transducin were also found to be antigenic targets in retinal autoimmune diseases (64, 65).

The numerous studies showed that GAPDH plays an essential role in the induction of autoimmunity, but how anti-GAPDH immune response originated in various diseases is still unknown. The autoimmune response against GAPDH might be initiated by a foreign, non-human, and GAPDH, which was found to be present on the surface of bacteria, viruses, and parasites subsequently cross-reacting with human protein (55). Cancer can also be a source of GAPDH because it was found to be overexpressed in many human cancer cells, including breast, prostate, pancreatic, lung, renal, gastric, liver, colorectal, bladder cancers, melanoma, and glioma (6, 66, 67). Those cancers are often associated with paraneoplastic syndromes, where antibody responses, initiated against tumor antigens, have adverse effects on distant tissue targets that have the same antigen or antigenic peptide (68, 69). The occurrence of AAbs specific to enolase, aldolase C, and GAPDH, was two to three times more frequent in CAR with gynecological cancers than in healthy women (59). Anti-GAPDH AAbs were also reported in 47% of patients with SLE, a chronic autoimmune disease that can damage any part of the body (skin, joints, and other organs) (55).

## Aldolase

Aldolase C has also been identified as an important autoantigen. In glycolysis, it catalyzes the reversible conversion of fructose-1,6-bisphosphate to glyceraldehyde 3-phosphate and dihydroxyacetone phosphate (Figure 1). However, similar to the other enzymes, aldolase also plays several non-glycolytic roles and interacts with vacuolar-ATPase and other molecules (70). The aldolase isozyme family is composed of three members, A, B, and C, which are encoded by separate genes. The proteins are expressed in a tissue-restricted manner (71, 72). Aldolase A is expressed predominantly in muscle and brain, B in the liver, and C in the brain and retina. Neuronal expression of aldolase C has been reported in the cerebellum Purkinje cells and in all cell types of the retina (21, 72). The highest expression was shown in the ONL of the retina, where cone and rod photoreceptors are aligned, and in the inner nuclear layer (INL), consisting of the bipolar, horizontal, and amacrine neurons, as well as in Müller glia cells (72).

Aldolase C interacts with GAPDH and catalyzes GAPDH inactivation in the presence of extracellular signals, indicating that such a complex has a regulatory function (73). In addition, aldolase strongly interacts with cytoskeletal elements. Aldolase A has also been found in the nucleus of many types of tumors, and is involved in the regulation of cell proliferation (74, 75).

The occurrence of AAbs against aldolase was associated with various autoimmune diseases and cancers (4, 76, 77). Anti-aldolase A and C AAbs have been shown in CAR (59), MAR (78), and diabetic retinopathy (19). The association of serum anti-aldolase AAbs was reported in age-related macular degeneration (AMD), a degenerative disorder of the central retina (macula), suggesting that an increased presence of such antibodies could lead to the disruption of aldolase functions and inflammation in the retina (79, 80). Anti-aldolase A AAbs were also present in sera of patients with the most common human neurodegenerative disease, AD, suggesting their potential role in development of this disorder (81). They were also found in patients with hyperkinetic movement disorders and Parkinson’s disease, but were not detectable in individuals with other inflammatory and non-inflammatory central nervous system diseases (82). Increased glycolytic activity and the presence of AAbs have been found to be related to tissue destruction of synovium in RA (83). Glucose phosphate isomerase, enolase, and aldolase are the key enzymes that promote RA autoimmunity by acting as target autoantigens, especially in early, untreated RA (5, 84). Ukaji et al. detected anti-aldolase A AAbs in RA patients with severe bone erosion, and suggested that a certain event may promote the production of AAbs by exposing hidden epitopes of aldolase A to the immune response, thus leading to the production of antibodies (83).

## Pyruvate Kinase (PK)

Pyruvate kinase catalyzes the last step of glycolysis and, similarly to other enzymes, is expressed in different isoforms, depending on tissue metabolism (85). PKM1 and PKM2 are expressed in cancers and normal tissues (86–88). PK is a multifunctional protein too, participating in a variety of pathways, protein–protein interactions, nuclear transport, metabolism reprogramming, gene transcription, and cell cycle progression (85). In the retina, PKM1 exists in the Müller cells and neurons, and PKM2 in glial cells and rod and cone photoreceptors, where it was found to co-localize with rhodopsin (89). PKM2 has generated a lot of interest due to its impact on changes in cellular metabolism observed in cancer, as well as in activated immune cells, by controlling activity of hypoxia-inducible factor 1-alpha and STAT3 during inflammation (90). As in other glycolytic enzymes, PKM2 was found to be an antigenic target in AR, as well as in other neurological conditions and cancers (6, 79, 80). PKM2 was targeted by AAbs in both geographic and neovascular AMD, but the level of anti-PKM2 autoantibody was best correlated with the early stages of AMD (79). PKM1 was identified as an autoimmune target in Tourette syndrome and associated neurological disorders (91). Moreover, anti-PKM1 AAbs reacted strongly with surface antigens of infectious strains of streptococcus, and antibodies against streptococcal M proteins reacted with PK, suggesting that anti-PK antibodies originated from streptococcal infection. Furthermore, anti-PKM1 autoreactivity was significantly higher in patients who had recently acquired a streptococcal infection with exacerbated symptoms, as compared to patients with exacerbated symptoms but no evidence of a streptococcal infection (91). In spite of association of serum anti-glycolytic enzyme AAbs with many of those diseases, it is not clear whether such AAbs are the direct result of, or are made due to the released antigenic proteins from damaged cells, including failing photoreceptors in the retina during progression of macular degeneration in AMD or AR.

## Anti-Glycolytic Antibodies in Cancer—Paraneoplastic Syndrome

Autoimmune disorders associated with cancer have been described as paraneoplastic complications in a distant organ, such as the retina (92). Paraneoplastic retinopathies are rare disorders associated with cancer, not caused by cancer invasion or metastasis or are consequences of treatment. In CAR, they may result in rapid and complete blindness. It is most commonly associated with small cell carcinoma of the lung, breast, and gynecologic cancers, but associations with lymphomas, non-small cell lung, prostate, pancreatic, bladder, and colon cancers have been described (15). The generation of AAbs during tumor formation, in response to aberrant cancer antigens, is the proposed mechanism of the CAR and MAR syndromes (26, 93). CAR-like symptoms can precede the manifestations of cancer. A strong similarity or identity of autoantigens associated with AR and cancer suggests that initially, AAbs may originate against tumor antigens during tumorigenesis, and then, after crossing the blood–retinal barrier (BRB) from the circulation, and accessing retinal cells, they cross-react with remote retinal antigens (24–26, 94–96). Although healthy individuals may have AAbs against these enzymes, they are likely to be natural antibodies, primarily polyreactive IgM, which display a moderate affinity for antigens or anti-microbial antibodies lingering after infections (92, 97). Serum IgG subclass distribution and levels in patients with autoimmune diseases differs from that in healthy people (93). In AR, the target autoantigens are protein antigens, T cell-dependent antigens that can stimulate a generation of IgG1- or IgG3-AAbs. We do not know what factors (secretion of cytokines, etc.) can cause anti-retinal AAbs to become pathogenic. Moreover, the presence of an intact BRB, as well as brain–blood barriers combined with the unique microenvironment of the eye or brain, ensures that the immune attack is weakened (94). IgG might reach intra-retinal structures through breaches in the BRB or from receptor-mediated uptake. IgG is also present in the healthy eye at very low levels relative to plasma levels without harmful effects (95). In contrast, long-lasting, high-affinity AAbs of the IgG class show pathologic properties related to alterations in cell clearance, antigen-receptor signaling, or cell effector functions (96).

A common feature of solid tumors is an increased aerobic glycolysis to generate ATP (the Warburg effect) (98). The genes encoding glycolytic enzymes are overexpressed in the majority of clinically relevant cancers, particularly genes encoding ALDO, ENO1, GAPDH, and PKM2 (15, 99, 100). Moreover, these enzymatic proteins play sometimes similar cellular roles in their non-glycolytic capacity, e.g., GAPDH activates survival pathways, enolase controls transcriptional regulation, aldolase promotes epithelial mesenchymal transition, and PKM2 enhances transcription and stabilization (100, 101). As a result, differential overexpression of glycolytic enzymes and their exposure on the cell surface, cell turnover, and apoptosis, or their release into the extracellular environment, could initiate autoimmune responses and the production of specific AAbs (102).

## Pathogenic AAbs

The highly conserved structure of glycolytic enzymes and their ubiquitous presence in all tissues support strong antigenicity (enolase, aldolase, GAPDH). Most disease-related AAbs are IgGs that are somatically mutated, suggesting that helper T cells drive the autoimmune B cell response, including anti-enolase AAbs in CAR patients (24, 103, 104). AAbs against glycolytic enzymes can also be produced in response to their mutations, misfolding, degradation, overexpression in the cell, and the protein release from damaged tissue (21). These AAbs can target any retinal cell type containing an antigen, including photoreceptor cells, ganglion cells, or bipolar cells and cause retinal dysfunction. The high sequence homology between microbial enolase, aldolase, GAPDH and PK, and human proteins was likely to facilitate the initiation and development of autoimmune reactions when these proteins are expressed on the membrane (21, 49, 105). Therefore, a *molecular mimicry* has been proposed as a mechanism of AAb formation and a contributor to the pathogenesis of autoimmune disease, for example, AAbs present in post-streptococcal infections of CNS diseases (21, 106, 107).

Anti-glycolytic enzymes AAbs were mostly studied in association with autoimmunity because their serum prevalence was *not* strictly disease-specific, many investigators dismissed their pathogenic role. However, the lack of disease restriction of the AAb response to one disease may be related to an increased expression of glycolytic proteins in various organs that triggers an autoimmune response and the occurrence of AAbs with the same specificity in several chronic and autoimmune disorders (3, 21). The presence of AAbs to distinct epitopes within an autoantigen can be a sign of disease-specific pathogenic immune activity, while the recognition of multiple epitopes within the same autoantigen may not be disease-specific (108, 109). We can speculate that the reactivity to a particular autoantigen does not necessarily cause disease, but the presence of destructive AAbs of limited epitope-specificity can ultimately spread pathogenic autoimmunity (110).

An important question is whether the widespread presence of anti-enolase, aldolase, GAPDH, and PKM2 and possibly against other enzymes like phosphoglycerate mutase, alpha-enolase, triose-phosphate isomerase, and malate dehydrogenase in various conditions is a sign of their causal role and pathogenic activity (4). In the case of anti-glycolytic protein AAbs, the induction of pathogenic effects could be a consequence of destabilized production of energy and glucose use (2, 74, 101). The proposed pathogenic involvement of AAbs is based on several observations summarized in Table 1. First, the persistence of high-affinity anti-enolase, anti-aldolase, anti-GAPDH, and anti-PKM2 AAbs over the course of autoimmune and inflammatory diseases reflects their pathogenic involvement as compared to antibodies of healthy controls (4, 96). Second, specific AAbs are associated with disease progression and prognosis (36, 59, 104), e.g., PKM2 correlates with the severity and progression of AMD, suggesting their pathogenic association (79). Third, studies show that antibodies can penetrate the living cell and induce cytotoxicity *in vitro* (43). Fourth, antibodies have the ability to induce cell death by apoptosis (2). Fifth, antibodies have the capacity to induce tissue pathology *in vivo* as shown by active immunization with enzymatic antigens and by passive transfer of antibodies (111). Sixth, antibodies have the ability to inhibit the catalytic function of glycolytic enzymes. For instance, anti-enolase antibody significantly decreased the catalytic activity of enolase, which resulted in a depletion of glycolytic ATP and an increase in the intracellular calcium, leading to cell apoptosis (2). In MS patients, the percentage of anti-GAPDH AAbs in the CSF was significantly higher than in patients with other neurologic diseases (61). Such AAbs strongly inhibited the catalytic function of GADPH, which could be reversed by their pre-adsorption with immobilized enzyme (112). Thus, an increased intrathecal production of anti-GAPDH AAbs may lead to their binding of GAPDH present in axons and neurons, inhibition of GAPDH glycolytic activity, neuro-axonal apoptosis, and cytotoxicity. Also, in enzymatic assays, anti-aldolase AAbs of AD inhibited the aldolase enzymatic activity (81). All of these findings suggest that AAbs can adversely contribute to retinal and neuro-axonal degeneration.

**Table 1:** **Widespread occurrence of autoantibodies (AAbs) against glycolytic enzymes with pathogenic properties in autoimmune diseases**.

Pathogenic potential of AAbs	Potential pathological/significance	Antigen	Reference
Association with disease	Higher levels of AAbs in patients as compared with health individuals: – Cancer-associated retinopathy – Melanoma-associated retinopathy – Non-paraneoplastic autoimmune retinopathy – Age-related macular degeneration – Glaucoma – Inflammatory bowel disease – Rheumatoid arthritis – Alzheimer’s disease – Multiple sclerosis – Diabetic retinopathy – Systemic lupus erythematosus – Coronary heart disease	Enolase Aldolase glyceraldehyde-3-phosphate dehydrogenase (GAPDH) Pyruvate kinase M2 (PKM2)	(2, 4, 5, 10, 18, 23, 24, 31–33, 37, 38, 40, 49, 52, 54, 71–73, 75, 79, 83, 103)
Induction of cytotoxicity	– Intracellular antibody penetration and cytotoxicity *in vitro* – Inhibits cell proliferation	Enolase	(1, 2, 40)
Triggering cell death	Ability of antibodies to induce cell death by apoptosis	Enolase GAPDH Aldolase	(2, 19, 75, 112, 113)
*In vivo* functional effects	Decrease responses in electroretinogram, retinal cell dysfunction	γ-Enolase (NSE) α-Enolase	(43, 103, 110, 114)
Inhibition of enzymatic activity	– Anti-enolase AAbs inhibited catalytic function of enolase – Anti-GADPH AAbs strongly inhibited the catalytic function of enzyme – Anti-aldolase AAbs inhibited the aldolase enzymatic activity	Enolase GAPDH Aldolase	(2, 75, 99)
Overexpression in tumors	Lung, mammary gland, prostate, lymph node, cervix, cartage bone marrow, brain, colon, liver, and thyroid	Enolase Aldolase GADPH PKM2	(57, 60, 78, 86, 87)

We have identified α-enolase as a target autoantigen in CAR, MAR, and AR (24, 31, 78). Seropositive patients have a worse prognosis than seronegative patients. Patients with AAbs had more abnormalities in the rod and cone photoreceptor function, as confirmed by ERG, than seronegative patients (104). In particular, the loss of central vision was more evident and more frequent in anti-enolase seropositive patients (104). Our research showed that anti-enolase antibody played a pathogenic role in retinal cell survival and determined the molecular events occurring before and during the induction cell death induced by antibodies (1, 2, 43, 113). When anti-enolase antibodies were cultured with retinal cells *in vitro* they triggered an apoptotic cell death, as examined by morphological changes and presence of TUNEL-positive cells (1). Cytotoxic effects induced by anti-enolase autoantibody appear to be specific, since normal IgG added to the culture at the same amount did not cause cell death. This apoptotic effect is similar to the action of anti-recoverin AAbs on E1A.NR3 retinal cells (114). Internalization of anti-recoverin IgG antibody or its Fab fragments by retinal cells mediated by endocytosis leads to cytotoxicity (115).

Treatment of living retinal cells with anti-enolase antibody-induced considerable changes in the ATP production, decrease in intracellular pH, and increase in intracellular calcium levels, which led to their apoptotic death (2). Retinal cells could be protected from anti-enolase antibody-induced apoptosis *in vitro* by resveratrol, a natural plant-derived drug, through multiple early molecular processes, such as reduction of intracellular calcium levels, down-regulation of Bax, upregulation of Sirt1 and Ku70 activities, and inhibition of caspase-3 activity (116). In the retina, antibodies to α-enolase mostly labeled the retinal ganglion cells and INL cells (43). Using *ex vivo* experiments and intravitreal injections, we showed that such antibodies were capable of penetrating retinal tissue and targeting the ganglion cells and INLs, and subsequently inducing their apoptotic death (43). Animal experiments have shown that an intravitreal injection of serum specific for enolase or purified anti-enolase antibody caused functional changes in the retina, showing reduced b-wave amplitudes as recorded by ERG (111, 117). Anti-α-enolase AAbs and autoantibody against γ-enolase (NSE) that were found in glaucoma, induced retinal dysfunction *in vivo* in a similar fashion to the effects induced by *N*-methyl-d-aspartate (117, 118). These findings showed that anti-enolase antibodies can play a causative role in the induction and progression of retinal degeneration in animals (30, 43, 59, 119). Antibodies that recognize and bind to cell debris in subretinal space during retinal degeneration may also trigger inflammation and synthesis of more AAbs (120). AAb binding to the cell surface-exposed enolase has led to opsonization or cell destruction, an increased inflammatory reaction, and in effect, tissue damage (121, 122). Taken together, anti-enolase AAbs have a potential to induce retinal degeneration, not only by the local formation of immune complexes but also by the direct damage to retinal cells and influence their cell function.

## Management of Autoimmune Retinopathies

Current therapies of CAR, MAR, or AR include systemic immunosuppression with steroid, intravenous immunoglobulin (IVIg), plasmapheresis, cytotoxic medications, and rituximab. However, there is no one commonly accepted protocol, so our knowledge is limited. Early diagnosis followed by treatment of AR is important to prevent widespread retinal degeneration and permanent vision loss. Moreover, delayed initiation of treatment in the course of disease may lead to the poor visual prognosis. The most common treatment has been long-term immunosuppression with steroids (26). Short-term therapy can be done, such as intravitreal triamcinolone and subtenons depomedrol, but these do not treat the undelaying causes of this systemic autoimmune disease; thus, longer term immunosuppression may be a better approach (123).

The most common sources regarding AR treatment benefits are published case reports. For example, a brief course of oral corticosteroids in a patient with anti-enolase AAbs caused an improvement in visual fields, disappearance of enolase-α AAbs, partial recovery of the cone response, and complete recovery of the rod response as measured by ERG (124). These findings suggest a pathologic role for enolase-α AAbs in this autoimmune rod/bipolar cell dysfunction.

In another study, the authors report that anti-GADPH can cross the placenta (125). A seropositive patient with AR and severe myasthenia gravis (MG) experienced a rapid progression of vision loss from driving vision to the hand motion/light perception level over 2-year period (125). The patient then underwent weekly plasmapheresis therapy, which led to an improvement of her symptoms. She became pregnant during the course of treatment. During the time of delivery, peripheral blood was collected from mother, as well as umbilical cord blood. Both samples were seropositive for anti-GAPDH among other AAbs. Despite the presence of those AAbs in the cord blood, the 6-month old patient appeared to have developed normal visual function and no MG symptoms.

Intravenous immunoglobulin may be another treatment option in addition to corticosteroids or plasmapheresis that is offered to patients with paraneoplastic visual loss. IVIg infusion showed promising results in some patients. In one study of three seropositive patients with CAR, including anti-enolase AAbs treated with IVIg, some improvement in visual field and light perception was shown (126). In another case of a patient with MAR and worsening visual function, an infusion of IVIg was administered. Patient ERG responses were consistent with MAR but his antibody status was unknown [although patients with MAR may have anti-enolase AAbs (31)]. Over the course of treatment, the patient noted progressive improvement in night vision, peripheral vision, and photopsias (127).

The number of AR patients benefited from rituximab treatment (128–131). The efficacy of rituximab was studied in six patients (12 eyes) administrated as a monotherapy or in combination therapy (132). Some patients in the study had anti-enolase (three patients) and anti-aldolase (one patient) AAbs. Stabilization and/or improvement of visual acuity, visual field parameters, and electroretinography parameters were observed in 75% of patients treated with rituximab. CAR, MAR, and AR are characterized by persistently elevated levels of ant-retinal AAbs, therefore, B cell depletion therapy based on rituximab delivers promising therapy for those patients. In view of these findings, larger scale studies of should be pursued in the future.

## Conclusion

**Figure 3:**
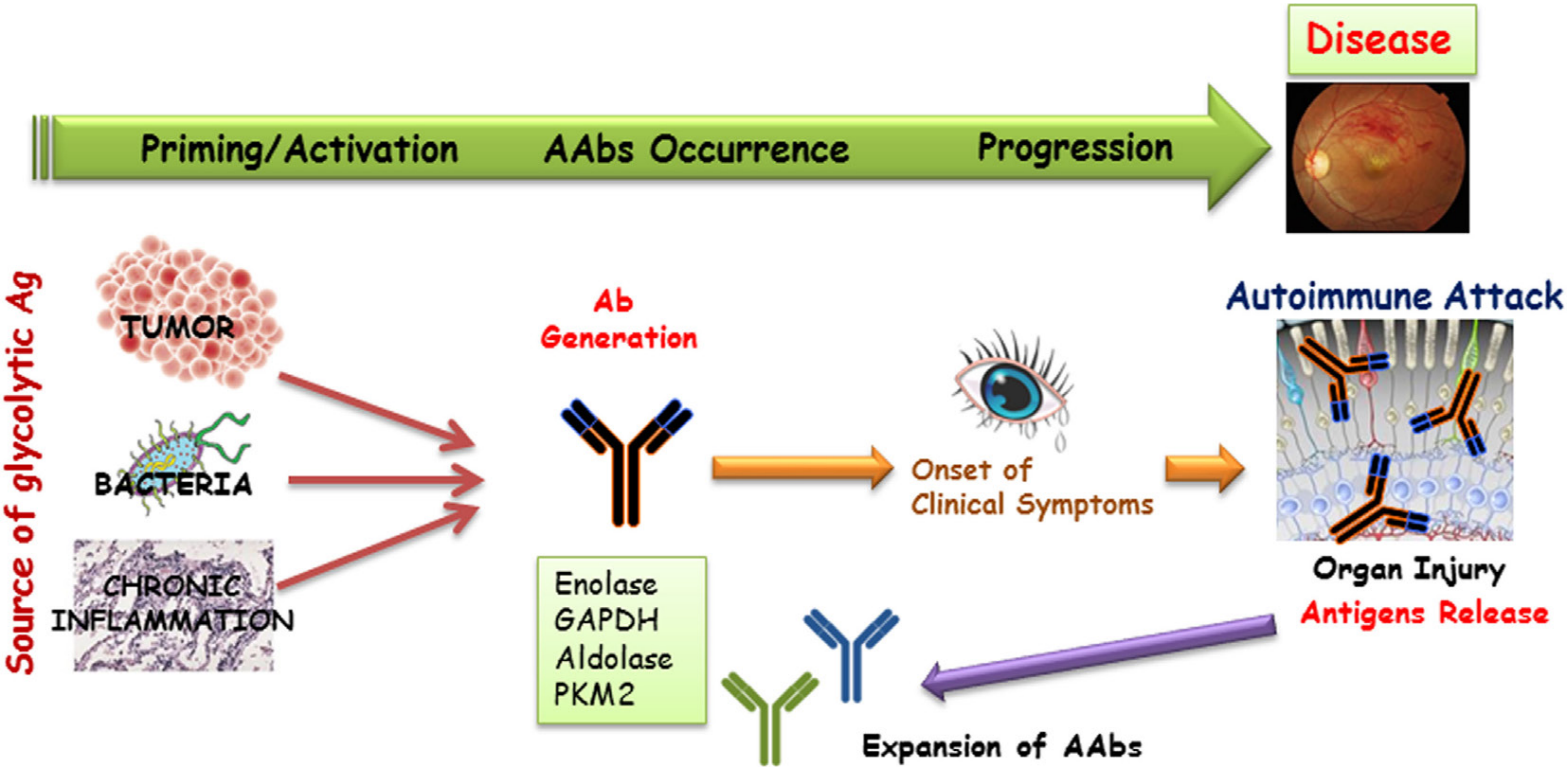
**A possible mechanism for the generation of anti-glycolytic proteins antibodies and their role in the induction of the retinal disease process**. Antibodies that are generated as a result of tumor, infection, or cell turnover during inflammation may target common antigens (the same specificity antigens) in the retina. Such antibodies can be present in the serum for a long time before clinical presentation of disease occurs. Degenerating tissue can be a source of new antigens that can initiate the further expansion of autoantibodies (AAbs) production and acceleration of disease.

Dissecting the possible role of anti-enzymatic protein AAbs with such a broad presence in AR and other diseases is a challenge. We propose that some specific AAbs may be unique to CAR and others may not. There are multiple mechanisms responsible for antibody generation, including the innate anti-microbial response, anti-tumor response, or autoimmune response against released self-antigens from damaged, inflamed tissue (Figure 3). AAbs target the same antigens as antibodies present in healthy subjects, but in low titers, lingering after infections. This suggests that at least some autoimmune diseases might emerge from a pathogenic shift in the phenotype from a normal stage to autoimmunity (109, 133). In other words, the occurrence of an autoimmune disease might not require a new autoimmunization, but rather a loss of control in the existing autoimmunity. The other possibility is that the presence of anti-glycolytic enzyme AAbs can represent a result rather than a direct cause (epiphenomenon). In such a scenario, dying photoreceptors by apoptosis, induced by some other mechanisms, produce substantial amounts of debris, containing high concentrations of the targeted antigens (glycolytic enzymatic proteins) released from OS, which could result in autoimmunization and enhanced permeability of the BRB (122). Thus, the serum presence of anti-glycolytic enzyme AAbs, whose importance had been previously dismissed, might be without pathological consequences until a failure of the BRB function, in effect allowing pathogenic AAbs access to their retinal targets, ultimately leading to damaging effects. Because of the presence of AAbs with several specificities in a single patient, this suggests that AAb arrays, rather than AAbs against a single antigen, might be responsible for degenerative processes in AR.

## Author Contributions

The author was totally the sole responsible for the design and writing of this review article.

## Conflict of Interest Statement

The author declares that the research was conducted in the absence of any commercial or financial relationships that could be construed as a potential conflict of interest.
